# Toward Sustainable Consumption Behavior in Online Education Industry: The Role of Consumer Value and Social Identity

**DOI:** 10.3389/fpsyg.2022.865149

**Published:** 2022-04-07

**Authors:** Songyu Jiang, Nuttapong Jotikasthira, Ruihui Pu

**Affiliations:** ^1^Rajamangala University of Technology Rattanakosin, Salaya, Thailand; ^2^Faculty of Economics, Srinakharinwirot University, Bangkok, Thailand

**Keywords:** sustainable consumption behavior, online education, psychology, quality education, value, identity

## Abstract

The prosperous development of online education in the digital age harvested countless consumers. Education for sustainable development is an important proposition for both academic community and practitioner, however, current little studies have shed light on Sustainable Consumption Behavior in online education industry (SCBOEI). The Consumer Value Theory and Social Identity Theory as theoretical basis linked with the field of Sustainable Consumption Behavior. This study is to further investigate the role of consumer value and social identity in the relation to Sustainable Consumption Behavior. Putting forward suggestions from this study to online education providers toward sustainable development.

**Method:**

Data on the impact of personal value on SCBOEI through a survey method with 552 valid students as respondents are collected from higher education institutions in China. A structural equation modeling approach is employed in this study for data analysis.

**Results:**

The result shows at the level of excellent model fit as indicated by all indicators: *X*^2^/DF = 1.053 (<3), RMSEA = 0.010 (<0.08), CFI = 0.991, GFI = 0.971, TLI = 0.989, AGF = 0.961 (>0.9). The results showed that, through social identity, functional value (indirect effect = 0086, *P* < 0.001), emotional value (indirect effect = 0061, *P* < 0.001), and social value (indirect effect = 0.073, *P* < 0.001) influence the variance of SCBOEI. The finding reveals that both theories can explain the SCBOEI of higher education students by showing that functional, social, and emotional values as well as social identity are powerful predictors of the Sustainable Consumption Behavior. The proposed model highlights the mediating role of social identity between SCBOEI and the three values. The functional, emotional, and social values influence SCBOEI directly and through social identity.

**Implications:**

The study significantly contributes to market promotion, college students, education planning, and teaching. Online education market personnel and college students can better understand the significance of sustainable development aspect of online education. Teaching and learning activities help lead students to SCBOEI by shaping their values and identities while paying more attention to quality education, knowledge sharing, and social equality.

## Introduction

**FIGURE 1:**
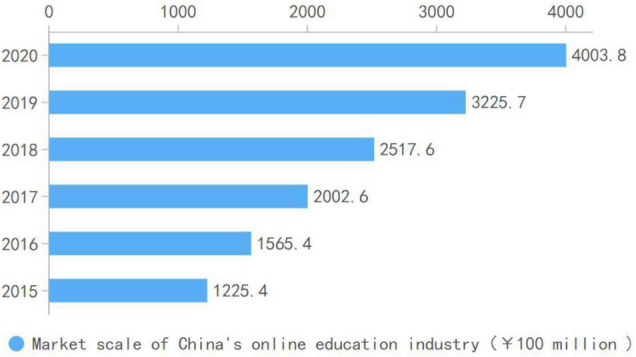
Scale statistics of online education market from 2015 to 2020 in China. Source: https://bg.qianzhan.com/trends/detail/506/210730-5d593518.html.

**FIGURE 2:**
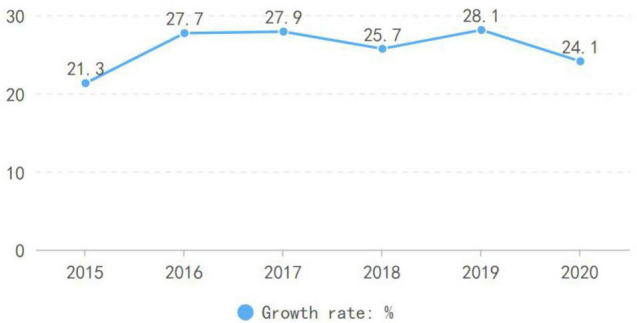
Growth rate of online education market from 2015 to 2020 in China. Source: https://bg.qianzhan.com/trends/detail/506/210730-5d593518.html.

Education for sustainability is a crucial word of global development strategy (Gadotti, 2008). More and more countries have committed to promoting sustainable development through education in order to realize their respective sustainable development goals (Bebbington and Unerman, 2018). The digital age has radically changed the educational model by closely integrating education to science and technology (Tan et al., 2021). Online education has become one of the “raison d’être” of education in the digital age and created much value for society (Resnick, 2002). COVID-19 seems to be a watershed in the development of online education as it replaces the traditional mode of instruction during the lockdown periods worldwide (Langlois et al., 2020). Online education plays an significant role and has made outstanding contributions to education development at all levels (Subotnik et al., 2011). As a part of the domineering service industry, online education has made essential breakthroughs in brand, customer, and sales in recent years. It has harvested many consumers from preschool to higher education, reiterating its significance to the development of the market economy. Many online education enterprises have attracted unexpected investments, and, consequently, have developed rapidly worldwide while creating an online education industry chain. Although in 2021, the Chinese government-controlled online subject education in the K12 stage to assure the quality and equity, the growth and development in terms of forms and quality still remain optimistic. With China’s emphasis on education, the national financial investment in education is increasing, the proportion of fixed capital in education is increasing annually, in that, online education has ushered in rapid development (Huo et al., 2021). Figure 1 depicts the market size of Chinese online education and its growth rate between 2015 and 2020. According to Figure 2, online education market has shown a continually strong growth rate (above 20 percent per annum) throughout the reported period with the market value of 4003.8 million Yuan in 2020. The sector proves to be not only resilient to fast changing socio-economic conditions but also a significant economic sector.

**FIGURE 3:**
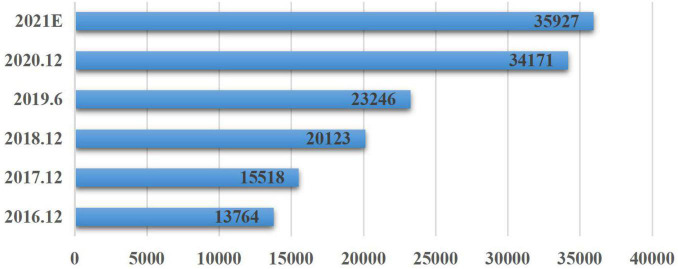
Scale and forecast of online education users in China from 2016 to 2021 (user scale: 10,000). Source: https://bg.qianzhan.com/trends/detail/506/210730-5d593518.html.

Figure 3 reports and estimates the market size of online education market in terms of users between 2016 and 2021. The number of users significantly increased yearly and is expected to exceed 35,927 million after 2021. Obviously, online education market in China is significant in terms of value, number of users and growth. There are apparent opportunities for the development of online education in China. In terms of segment, Higher education accounts for more than 30% of the online education market, including online education products focusing on vocational training, professional courses, and language training. While there are a lot of issues related sustainable that China needs to tackle, sustainable consumption behaviors should be educated to and nurtured in its young people. Evidently, online education, as a replacing mode of education to classroom encounters, is a critical point of social and economic development and other social activities. Under the challenge of a knowledge economy and digital age, the current research has abundant reasons to focus on SCBOEI.

Promoting Sustainable Consumption Behavior (SCB) is an important mean to achieve sustainable development goals (Figueroa-García et al., 2018). The energy industry, food industry, manufacturing industry, and agriculture are major scope of in the research on sustainable consumption behavior (Jiang and Pu, 2021). These studies usually explored the impact of value (Fischer et al., 2017), environmental attitude (Lee, 2014), identity (Kadic-Maglajlic et al., 2019), culture (Figueroa-García et al., 2018), media (Simeone and Scarpato, 2020), and religion on sustainable consumption behavior (Orellano et al., 2020). Discussions on sustainable consumption behavior in the education industry are still rare. The sustainable consumption behavior of the online education industry is different from that of other industries that mainly focuses on the environment (Pu and Jiang, 2021). SCBOEI concerns more about the realization of the sustainable development goals through quality education, social equity, health, economic development, innovation, sustainable cities and communities, and international relations (Jiang and Pu, 2022). As a new education model in the digital era, online education is critical for knowledge sharing, talent training, and lifelong education which are all the social side of sustainable development (Jiang and Pu, 2022). To present, there are still no concrete evidence on optimal measures and methods to use online education to serve sustainable development goals.

Education for sustainable development is public awareness-raising and sustainability-related training that enables learners to make informed sustainable choices in their lives (Pu et al., 2022). Online education for sustainable development can be provided at all levels from preschool education while gradually improving knowledge, skills, attitudes, and values conducive to sustainable development (Pu and Jiang, 2021). To reach at the desired goals in sustainable consumption behavior in learners, many factors must be taken into considerations not only education, but also other factors including value, identity, attitude, participation, knowledge, and even other situational factors (Jiang and Pu, 2021).

In order to realize the SCBOEI among learners, current research must understand the factors affecting the SCBOEI. Thus, it is necessary to understand the influences of value social identity on SCBOEI. In addition to putting sustainability in the development strategy of the online education industry, consumer must also improve their individual sustainable consumption behaviors. In that light, value and identity are essential factors determining sustainable consumption behavior (Lazaroiu et al., 2019). Unfortunately, the influences of such variables have not yet been explored in the context of education industry. Using Consumer Value Theory and Social Identity Theory to explore their influences on SCBOEI can fill the current research gap. There is an established conceptual framework on sustainable consumption behavior of the online education industry resulted from the impact of value and identity (Jiang and Pu, 2021). However, there is no empirical research to confirm the model. Based on this circumstances, quantitative methods are used to explore the impact of value and identity on SCBOEI help fill the current research gap. In other words, these social and psychological factors play an essential role in shaping sustainable consumption behavior for consumers in the online education industry. It is worthwhile to uncover which of them can really explain and predict sustainable consumption behaviors in online education contexts.

Principally, this study aims to explain the relationship of value, identity, and SCBOEI. The finding can further enlighten scholars and practitioners on the concept of SCBOEI and provide guidelines how to promote and nurture sustainable consumption behaviors in general as well as in education contexts.

## Theoretical Approach: Consumer Value Theory and Social Identity Theory

### Consumer Value Theory

Sweeney et al. (1997) postulated that consumer value could significantly explain consumer behaviors. Consumer value can be divided into three dimensions including functional value, social value, and emotional value (Sweeney et al., 1997). Functional value refers to consumers’ perception and evaluation of price and quality of a market offering (Sweeney and Soutar, 2001). Social value reflects a social image or self-image formed by consumers through consuming, acquiring, or possessing such offerings (Sweeney and Soutar, 2001). On the other hand, social value is reflected in a kind of social support gained from consumption, purchase or ownership of good or services in a particular context’s consumers confirm or disconfirm their consumption behavior choices (Brickson, 2007). Emotional value is a sense of pleasure and satisfaction. Consumers obtained from their behavioral choices and form a consumption behavioral trait consistent to the resulting emotional pleasure (Johnson and Stewart, 2017). The concept of consumer value should investigate the impact of these three values on the behavioral choices (Sheth et al., 1991). Thus, the research posits that functional value, social value, and emotional value are collectively important to predict SCBOEI.

*Functional value* (*FV*) refers to consumers’ judgment and understanding of functions, price and quality of products or services (Chen et al., 2005). In the online education context, functional value usually refers to the convenience of use and access and diversity of the program perceived by learners. College students believe that online education limits the negative impacts of learning time and space and help realize the wish of students who want to learn and explore certain subjects irrelevant to time and space (Major, 2010). Comparing to traditional mode of instruction, it provides more learning resources and more choices quality instructors (Leacock and Nesbit, 2007). Online education seems to jump out of the constraints of traditional face-to-face learning, make learning freer, and improve efficiency (Elumalai et al., 2021). As for the case of purchase behavior online education has a characteristic of one purchase and multiple consumption, allowing buyers to repeat the learning experiences or share with others (Turel et al., 2010). Simply put, online education offers a good value in terms of consumer cost as the consumer can repeat the content and revisit the topics they do not understand repeatedly and experience classes offered by different instructors (Pu et al., 2022). Online education is also an enabler of knowledge-sharing era as it allows learners to expose to and acquire knowledge contents they are interested in as well as discussing with other peer learners, instructors or experts in the topics at the same time. Online education is, therefore, tool facilitating the achievement of lifelong learning and comprehensive learning aspirations (Yuan et al., 2008).

*Emotional value* (*EV*), also known as a hedonic or experiential value, is the most significant to achieve a certain feeling or emotional state based on different types of values derived from consumption, ownership, and purchase of goods and services (Khan and Mohsin, 2017). Emotional consumption involves multiple senses, fantasies, emotions, holistically received from product and brand experience (Hollebeek et al., 2022). In this articles, emotional value in online education as the pleasure that consumers feel when purchasing and consuming online education products, which is the subjective to understanding, recognition, and acceptance of online education. The two aspects of emotional value include aesthetics and playfulness (Kato, 2021). The emotional value is derived from learners’ perceived interactiveness and comfort with online education (Jiang and Pu, 2021).

*Social value* (*SV*) refers to a psychological state that consumers perceive higher social recognition from their associate groups as a result of ownership, acquisition and consumption of goods and services (Wang et al., 2014). There is always a strong correlation between social value and sustainable consumption behavior, which is always linked to social, environmental, equity, and economic problems (Lazaroiu et al., 2019). Through sustainable consumption behavior, consumers contribute to the society in order to achieve social satisfaction (Wang et al., 2019). Due to peer pressure and group influences, SCBOEI is a kind of social behavior. The consumption process reflects symbolic meanings, social norms, associations, consumer identity, and self-positioning within a particular context. The social value of online education industry emphasizes a kind of social responsibility engaged by users during consumption (Pu and Jiang, 2021). It is believed that the consumption of the online education industry is derived from a mutually beneficial dialog between one’s self and his/her society, which can integrate itself into the circle of the digital age, keep up with the trend and tide of education development, recognize the future of online education development, and have a specific social foresight. Specifically, the social value of the online education industry offers articular symbolic or attention drawing benefits to learners (Jiang and Pu, 2022). For consumers, online education helps improve consumers’ self-concept and form a kind of consumption voucher with benign development (Taylor, 2020). Therefore, the social value in the online education industry can be regarded as an integration of learners’ social self-concept and social recognition from aspirational groups (Chen and Chen, 2020).

### Social Identity Theory

In exploring the sustainable consumption behavior, social identity theory has become one of adopted disciplines (Reyes-Menendez et al., 2020). Sustainable consumption behavior brings consumers an identity of being accepted by society allowing them to smoothly integrate into aspirational groups (Abdulrazak and Quoquab, 2018). This identification is because consumers’ perceived behavior in consumption can achieve particular social benefits, and further reinforces their f social self-concept (Abdulrazak and Quoquab, 2018). Social identity has three elements, cognitive, affective, and evaluative (Wang, 2017). Cognitive social identity refers to individuals’ perception that they belong to a particular social group (Miller et al., 2009). Affective identity involves the feelings associated with being a part of a particular social group (Belanche et al., 2017). Evaluative identity concerns with positive or negative judgments individuals make about the group they belong to (collective self-esteem) (Belanche et al., 2017).

The online education industry’s social identity (SI) can be defined as an identification with one’s group category partially through SCBOEI. Belongingness to certain groups influence one’s inclination toward SCBOEI. Specifically, consumers have formed a kind of social self-concept through the consumption of the online education as a result of perceived participation of digital trends and knowledge sharing. In other words, because consumers perceive that they have made certain contributions to society via online education consumption, such as knowledge sharing, promotion of educational equity and educational development. Through the consumption behavior, they help promotes economic development and knowledge dissemination, and technology development. Table 1 lists the definition of variables included in this study.

**TABLE 1:** Definition of variables in the study.

Variable		Definition
Value	Functional value	Consumers’ perception of the price utility and special quality of online education.
	Social value	Consumers’ self-image and social relationship network formed through online education consumption.
	Emotional value	Consumers’ sense of pleasure, comfort and satisfaction through consumption online education.
Social identity		Consumers’ social self-concept and trust derived from the consumption of online education.
SCBOEI		Consumers’ commitment to quality education, economic development and social stability resulted from online education, and to use online education as a tool for sustainable development.

*SCBOEI, Sustainable Consumption Behavior in Online Education Industry.*

Value and identity are two important psychological factors affecting sustainable consumption behavior, which frequently appear in studies in the field of consumer behavior. More importantly, individuals need to pay more attention to the intermediating role of identity in the relationship of value and sustainable consumption behavior (Shove, 2004). Different values shape different identities (Hitlin, 2003). Some consumption behavior intention guided by perceived value provides clues for us to study sustainable consumption behavior (Japutra et al., 2020). Based on the theoretical model, functional value can well predict identity in the category of consumer value. Therefore, the research puts forward:

H1. Functional value has a impact on SI.

Similarly, emotional value and identity belong to the scope of psychological discussion (Khan and Mohsin, 2017). Perceived value can form a certain consumer commitment. Based on the online education industry (Loureiro et al., 2016), the emotional experience consumers feel is conducive to them to obtain some recognition. The previous article continued to discuss that emotional value can predict identity, so research suggests:

H2. Emotional value has a impact on SI.

Social value and social identity are two closely related concepts (Fujita et al., 2018). Based on the consensus of social sustainable development, social value usually shapes a kind of social identity (Fujita et al., 2018). This conclusion has been proved in the research of organization management and sustainable consumption (Wang, 2017). Therefore, this paper makes a hypothesis:

H3. Social value has a positive impact on SI.

Some empirical studies support those individuals with functional and social values tend to demonstrate more sustainable consumption behaviors (Park and Lin, 2020). Some scholars explored the relationship between value and identity and sustainable consumption behavior (Jiang and Pu, 2021). Functional value affects consumers’ consumption behavior from two dimensions: price and value (Kato, 2021). In the sustainable consumption of food, automobile, and agriculture. In particular, the value of luxury goods has a significant impact on consumers’ behavior (Loureiro et al., 2020). Functional value has long been an important factor for consumers to change their consumption behavior (Jiang and Pu, 2021). Therefore, the study proposes:

H4. Functional value have a positive impact on SCBOEI.

With the humanization of market transactions, more and more consumers are concerned about the consumption experience, and emotional value is an important manifestation of the consumption experience (Khan and Mohsin, 2017). Therefore, in the research of sustainable consumption behavior (SCB), emotional value is also considered to be an important factor in the formation of sustainable consumption behavior in many fields. Based on this, the study made a hypothesis:

H5. Emotional value has a positive impact on SCBOEI.

Social value refers to consumers’ perceived contribution to society and social feedback in consumption activities (Park and Lin, 2020). The formation of this value is often reflected in reciprocal behavior, which affects consumers’ sustainable consumption behavior (Brickson, 2007). Based on this, the study made a hypothesis:

H6. Social value has a positive impact on SCBOEI.

Social identity theory insists on the shaping effect of identity on behavior (Fujita et al., 2018). In other words, social identity plays an important role in the research of sustainable development (Schmader and Sedikides, 2018). Many studies encourage to regard consumer identity as an important factor affecting stable consumption (Schmader and Sedikides, 2018). The sense of integration brought by social identity can promote consumers to further change their behavior and desire to obtain respect and support through their behavior (Wang, 2017). Therefore, the article puts forward:

H7. Social identity has a positive impact on SCBOEI.

The two dimensions of price and quality contained in functional value are closely related to consumer engagement and consumer behavior (Itani et al., 2019). Value can be directly or indirectly related to pro-environment behavior through identity (de la Luz Escalona et al., 2015). The environmental self-identity mediates the link between consumer bio-spheric values and pro-environmental intentions and behavior (Kautish and Sharma, 2018). Consumer identity mediates the relationship between consumer values and sustainable consumption behavior (Dermody et al., 2015). Therefore, in the context of online education industry, the impact of functional value on the sustainable consumption behavior of this industry may be mediated by identity:

H8. Functional value indirectly influences SCBOEI through social identity.

Dermody et al. (2018) also supported the mediating role of identity between social value and SCB as Individuals who identify with their community are more likely to engage in SCB (Dermody et al., 2018; Schaefer et al., 2020). Although the SCBOEI is different from SCB in other contexts, considerable number of studies showed the evidence of the intermediary role of identity between social value and sustainable consumption behavior, providing a theoretical basis for constructing the conceptual framework of the present study.

H9. Social value indirectly influences SCBOEI through social identity.

Jiang and Pu (2021) argued the vital role of identity in online education’s value and sustainable consumption behavior from their qualitative studies, a confirmatory study is still needed. In the online education industry, emotional value is a new, special and relaxed experience that users feel in the process of online education consumption (Jiang and Pu, 2022). The intermediary role of identity in emotional value and sustainable consumption behavior inspires the content that should be paid attention to in shaping sustainable consumption behavior in the business field. Consumers’ emotional value plays a positive role in shaping their social identity (Makransky and Lilleholt, 2018), which has a positive impact on sustainable consumption behavior. This conclusion may also exist in the online education industry. Therefore, the research hypothesis:

H10. Emotional value indirectly influences SCBOEI through social identity.

## Materials and Methods

### Sample

An Internet survey with 552 college students in China was conducted during the period of 1 month using convenient sampling method. Demographic. The sample is described using demographic factors including e gender, grade, education level, location, and types of ownership of the education institution. The sample included 306 men and 246 women, mainly in studying in the undergraduate level (only 4 respondents in the postgraduate level). Location wise, most respondents are from western and southern China (127 and 220, respectively), while 90 students from the East and 115 from the north. As for types of ownership of the school, respondents mainly study enroll in public higher education institutions (43.7%), while there are 190 and 121 participants from private and public-private schools representing 57.3% of the sample as reported in Table 2. Table 2 describes the specific information.

**TABLE 2:** Demographic statistical analysis.

Sex	Male	306	55.40%
	Female	246	44.60%
Grade	Grade 1	111	20.10%
	Grade 2	150	27.20%
	Grade 3	177	32.10%
	Grade 4	110	19.90%
	Over bachelor	4	0.70%
Education level	Under bachelor	73	13.20%
	Bachelor	475	86.10%
	Master	4	0.70%
Location	East China	90	16.30%
	South China	127	23%
	West China	220	39.90%
	North China	115	20.80%
Institution attribute	Private	190	34.40%
	Public	241	43.70%
	Private and public	121	21.90%

### Instrument

The survey questionnaire comprises six parts. The first part asked the respondents to report their respective demographic profiles (i.e., gender, grade, level of education, location, and ownership of the institutions). Other parts of the questionnaire probed the respondents on the following aspects: functional value, social value, emotional value, social identity, SCBOEI (Appendix 1). The instruments were translated into Chinese (and back-translated into English for validity verification). To ensure the structural validity of the questionnaire, a confirmatory factor was analyzed, and multiple indicators were considered. The goodness of fit indices, chi-square to degrees of freedom (*X*^2^/DF), compare the fitting index (CFI), approximate root means square error (RMSEA), 95% confidence interval (90% CI) of RMSEA.

#### Measurement of Variables

SCBOEI is the dependent variable in this study. Three items iterating consumption phases, consumption areas, and sustainable consumption habits were used to measure respondents’ SCBOEI by adapting from questions adapted from the works of Biswas (2017) and Wang et al. (2014) on sustainable consumption behavior.

Independent variables of the proposed model include value and social identity. Eleven items were used to measure respondents’ values, by using four items measured functional value perceived by respondents about online education products adapted from the work of Biswas (2017). Emotional value was measured in 3 items adapted from the work of Lin and Huang (2012). Social value was measured in four items adapted from the work of Lin and Huang (2012). All items were adapted to fit to the context of online education. Six items were used to measure using 6 items on social identity adapted from the work of de la Luz Escalona et al. (2015) to fit to the study’s context. Table 3 summarize the variables and their respective sources. Appendix 1 list the detail.

**TABLE 3:** Source of variables measurement.

Variables	References
SCBOEI	Wang et al., 2014; Biswas, 2017
Emotional value	Lin and Huang, 2012
Functional value	Biswas, 2017
Social value	Lin and Huang, 2012
Social identity	de la Luz Escalona et al., 2015

### Data Analysis

Data collected from the survey was processed using packaged statistical program to obtain the mean score of composite variables, their respective indicators for validity and reliability. The primarily processed data were further analyzed to test the proposed hypotheses through path analysis and regression and finally Structural Equation Model -SEM. Structural equation model is an effective tool to test and retest hypothesis while controlling measurement errors (Wang and Rhemtulla, 2021). Firstly, the measurement model was tested, followed by confirmatory factor analysis (CFA) to evaluate the structural validity of the measurement. Next, SEM was tested to test hypothetical relationships proposed in the model. When evaluating model fitting, various fitting indicators (i.e., *X*^2^/DF, CFI, GFI, AGFI, RMSEA) were used. Finally, the bootstrapping method was conducted to test the mediating role of social identity as appeared in the model.

## Results

### Descriptive Results

Table 4 describes all items’ mean scores and their standard deviations. The average value of each item in SCBOEI is almost around 3, indicating that college students generally have a neutral understanding of the SCBOEI. Among other variables, the mean scores of emotional value and social identity values are relatively high, but the degree is not apparent. That means that college students generally do not clearly understand the influencing factors on SCBOEI. Whether it is functional value, emotional value, social value, or identity, the convergence of mean and standard deviation means the consistency of college students in dealing with this topic.

**TABLE 4:** Descriptive statistics of instruments used to explain sustainable consumption behavior in online education industry.

		Mean	Std. deviation
SCBOEI	Q6	2.90	1.236
	Q7	3.00	1.245
	Q8	2.99	1.259
Functional value	Q9	2.89	1.201
	Q10	2.96	1.169
	Q11	2.97	1.259
	Q12	2.99	1.204
Emotional value	Q13	2.96	1.168
	Q14	3.00	1.271
	Q15	3.01	1.238
Social value	Q16	2.88	1.217
	Q17	2.92	1.189
	Q18	2.90	1.239
	Q19	2.85	1.166
Social identity	Q20	2.96	1.198
	Q21	2.90	1.212
	Q22	2.91	1.236
	Q23	3.06	1.267
	Q24	3.05	1.242
	Q25	3.07	1.215

### Confirmatory Factor Analysis

In the Confirmatory Factor Analysis (CFA), *X*^2^/DF, RMSEA, CFI, GFI, TLI, and AGFI are important indicators of the model fit. All items and variables were tested by confirmatory factor analysis, showing the model fit as indicated by all indicators: *X*^2^/DF = 1.053 (<3), RMSEA = 0.010 (<0.08), CFI = 0.991, GFI = 0.971, TLI = 0.989, AGF = 0.961 (>0.9). Table 5 shows that an excellent model fit.

**TABLE 5:** Reliability and validity of the measurement model.

*X*^2^/df	RMSEA	CFI	GFI	TLI	AGFI
1.053	0.010	0.991	0.971	0.989	0.961

Table 6 reports significant inter-item correlations of all observed variables belonging to each of the latent variables.

**TABLE 6:** Regression weights in CFA.

			Estimate	*S.E*.	C.R.	P
Q6	←	SCBOEI	1			
Q7	←	SCBOEI	0.901	0.148	6.079	***
Q8	←	SCBOEI	1.021	0.157	6.511	***
Q9	←	FV	1			
Q10	←	FV	0.992	0.16	6.216	***
Q11	←	FV	1.065	0.172	6.203	***
Q12	←	FV	0.947	0.159	5.95	***
Q13	←	EV	1			
Q14	←	EV	1.024	0.168	6.111	***
Q15	←	EV	1.023	0.165	6.202	***
Q16	←	SV	1			
Q17	←	SV	0.879	0.153	5.724	***
Q18	←	SV	0.918	0.16	5.733	***
Q19	←	SV	1.105	0.169	6.551	***
Q20	←	SI	1			
Q21	←	SI	1.038	0.208	4.991	***
Q22	←	SI	1.305	0.236	5.523	***
Q23	←	SI	1.421	0.251	5.662	***
Q24	←	SI	1.356	0.242	5.601	***
Q25	←	SI	1.321	0.236	5.591	***

*** means the relationship between the variables is significant.

Table 7 shows significant correlations between latent independent variables and latent dependent variables, meaning that the correlations between functional value, social value, emotional value, social identity, and SCBOEI are significant.

**TABLE 7:** Results of the correlation analysis.

			Estimate	*S.E*.	C.R.	*P*
FV	↔	EV	0.211	0.039	5.408	***
FV	↔	SV	0.213	0.039	5.416	***
SCBOEI	↔	FV	0.231	0.042	5.535	***
FV	↔	SI	0.174	0.035	5.031	***
EV	↔	SV	0.223	0.041	5.482	***
SCBOEI	↔	EV	0.212	0.041	5.232	***
EV	↔	SI	0.172	0.034	5.017	***
SCBOEI	↔	SV	0.213	0.04	5.281	***
SV	↔	SI	0.167	0.034	4.945	***
SCBOEI	↔	SI	0.204	0.039	5.202	***

*** means the relationship between the variables is significant.

### Path Diagram

Significant relationships between all independent’s variables (functional value, emotional value, social value, and social identity) are found. The values of residual terms in the path graph are positive numbers, e1 = 0.31 and e2 = 0.25, respectively, and all non-standardized coefficients are positive numbers, meaning that the fitting degree of the model is appropriate and does not violate our estimation. Figure 3 shows the standardized estimated path coefficients of this study.

**FIGURE 4:**
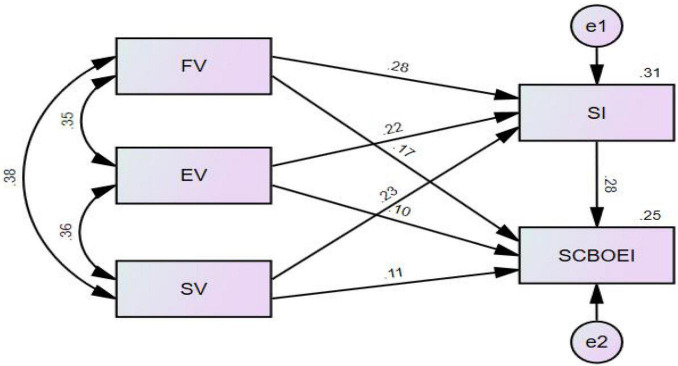
Standardized estimated path coefficient.

From Figure 4, all factors are suitable for further path analysis as Pearson correlation coefficients of each value does not indicate high collinearity problem (the correlation coefficients are lower than 0.70 critical value) (Purwanto and Sudargini, 2021). Therefore, functional value, emotional value, and social value are suitable to continue the path analysis.

C.R. usually greater than 3.25, *P*-value will be less than 0.001. In SEM, if the *p*-value is less than 0.001, it will be represented by * * *, which means that the relationship is significant (Mueller and Hancock, 2019). Generally, *P* < 0.05 indicates that the relationship is significant (Mueller and Hancock, 2019), however, for SEM, it indicates that the hypothesis is rejected. From the below table, all types of values have significant positive relationship with Social Identity. Among the three types of values, functional value expresses strongest relationship with Social Identity (*S.E*. = 0.036, CR = 7.058, *P* < 0.001) followed by social value (*S.E*. = 0.036, C.R. = 5.873, *P* < 0.001) and emotional value (0.032, C.R. = 5.589, *P* < 0.001).

All four independent variables have also shown significant positive relationships with the dependent variable- SCBOEI despite some reservations for two latent variables- emotional and social values. Among them, social identity has the strongest significant positive relationship with SCBOEI (*S.E.* = 0.055, C.R. = 6.22, *P* < 0.001), followed by functional value (*S.E*. = 0.048, C.R. = 3.87, *P* < 0.001). Although the correlations between SCBOEI and social value (*S.E.* = 0.048, C.R. = 2.679, *P* = 0.007, >0.001, <0.05) and emotional value (*S.E*. = 0.042, C.R. = 2.368, *P* = 0.018, >0.001, <0.05) do not meet the 0.001 significant assumptions, they still satisfy the 0.05 critical value for significance relationship. Based on the relationships reported in the Table 8, all hypotheses are confirmed.

**TABLE 8:** Path coefficient estimate of the final model.

			Estimate	*S.E*.	C.R.	*P*	Results	Hypothesis
SI	←	FV	0.251	0.036	7.058	***	Support	H1
SI	←	EV	0.178	0.032	5.589	***	Support	H2
SI	←	SV	0.211	0.036	5.873	***	Support	H3
SCBOEI	←	SI	0.344	0.055	6.22	***	Support	H7
SCBOEI	←	FV	0.187	0.048	3.87	***	Support	H4
SCBOEI	←	EV	0.101	0.042	2.368	0.018	Support	H5
SCBOEI	←	SV	0.129	0.048	2.679	0.007	Support	H6

*** means the relationship between the variables is significant.

In order to test the mediating role of social identity between the three types of values and SCBOEI proposed in the model, bootstrapping analysis was conducted. The results showed that, through social identity, functional value (indirect effect = 0086, *P* < 0.001), emotional value (indirect effect = 0061, *P* < 0.001), and social value (indirect effect = 0.073, *P* < 0.001) influence the variance of SCBOEI.

To determine understand the actual effect of each type of value to SCBOEI both through their respective direct effect and indirect effects through SI, total effects should also be considered.

Table 9 suggests the total effect of FV → SI → SCBOEI in the model is 0.273, and the proportion of intermediary effect is 0.316. The total effect of the path of EV → SI → SCBOEI in the model is 0.162, and the mediation effect accounts for 37.8%. The total effect of SV → SI → SCBOEI in the model is 0.201, and the proportion of intermediary effect is 0.36. By comparing the mediating effects of paths, the results of diff1 and diff2 are positive, and their estimates are 0.025 and 0.014, respectively, which means that the mediating effect of social identity on SCBOEI is stronger in functional value than in emotional value. Similarly, the mediating effect of social identity on SCBOEI in functional value is more potent than that in social value. However, the estimated value of diff3 is negative, which means that the mediating effect of social identity on SCBOEI is more potent than that of emotional value on SCBOEI. Therefore, the mediating effect of social identity on SCBOEI is the strongest in functional value, followed by the mediating effect of social value on SCBOEI, and the smallest is the mediating effect of emotional value and SCBOEI.

**TABLE 9:** Results of mediation analysis using bootstrapping.

	Path	Estimate	Lower	Upper	*P*
Indirect effect	FV → SI → SCBOEI	0.086	0.053	0.136	0.000
	EV → SI → SCBOEI	0.061	0.035	0.098	0.000
	SV → SI → SCBOEI	0.073	0.041	0.115	0.000
Total effect	FV → SI → SCBOEI	0.273	0.182	0.369	0
	EV → SI → SCBOEI	0.162	0.072	0.258	0.002
	SV → SI → SCBOEI	0.201	0.094	0.302	0.001
Mediation/total effect	FV → SI → SCBOEI	0.316	0.187	0.558	0
	EV → SI → SCBOEI	0.378	0.186	0.856	0.003
	SV → SI → SCBOEI	0.36	0.183	0.799	0.001
Mediation difference	Diff1	0.025	−0.013	0.072	0.199
	Diff2	0.014	−0.026	0.062	0.494
	Diff3	−0.011	−0.053	0.027	0.542
Diff indicates the difference of mediating effects of different paths diff1 = FV → SI → SCBOEI-EV → SI → SCBOEI diff2 = FV → SI → SCBOEI-SV → SI → SCBOEI diff3 = EV → SI → SCBOEI-SV → SI → SCBOEI					

To sum, all these results show that functional value, emotional value, social value, and social identity are directly related to SCBOEI, and they three types of value indirectly affect SCBOEI through social identity. Hence, mediation assumptions are supported, and the causal chain model applies to the model. Consequently, H8, H9, and H10 are supported.

## Discussion and Implications

The results of this study provide theoretical and practical significance. For academics, Consumer Value Theory and Social Identity Theory are still valid in predicting sustainable consumption behaviors, including social equity, knowledge sharing and awareness of the importance of sustainable development (Jiang and Pu, 2021). The two theories can be used in combination to explain and predict sustainable consumption behavior of Chinese college students in online education industry. This present study is also different from other academic works in the area of sustainable consumption in that it argues the causal chain with social identity plays a central role in determining the intended SCBOEI.

For practitioners, despite unclear understanding about the role of functional, social, and social values as well as social identity on sustainable consumption behavior. However, those who perceive high level of functional value of online education and identify themselves with online education community are inclined toward more sustainable consumption behaviors in the online education industry. The finding suggests that Chinese college students still lack the awareness of sustainable consumption behavior in the online education industry especially in the aspect of social equity and knowledge sharing. The finding indicates a necessity for all concerned parties of the online education industry to work more toward sustainable development goals.

Nevertheless, college students are optimistic about the sustainable consumption behavior of the online education industry thanks to positive interpretation of value and high level of self-identification. Sustainable consumption behavior inclination among college students might be a result of the work of various concerned parties including their pre-college education, government policies, market environment, and other factors. For them online education products are just a market offering they consume. It is imperative to build the relationship between their consumption behavior and social responsibility. In addition, college students’ consumption of online education products shows more demand for functions due to the nature of educational products and private consumption contexts. Higher awareness of social aspects of sustainable development and potential contributions of online education might help students engage more in SCBOEI.

The SEM analysis in this study explained positive causal relationship between value, identity, and SCBOEI. In previous studies, the functional convenience, content richness, and sharing of online education have promoted the behavior of online education (Tseng and Wei, 2020). Value and identity serve as inherent motivation to adopt online education (Eccles and Wigfield, 2020). In other words, when people feel the value of consuming online education, both functional, social, and emotional values, they have a higher predisposition to consume online education products. In this sense, it is necessary to establish a model of value conducive to the sustainable consumption behavior of the online education industry by enhancing learners’ identification and the perception of both functional and psychological values.

Similar to other sector, functional, social and emotional values are salient factors influencing the consumption behavior in the online education industry. Relative stronger relationships between perceived functional value and social identity of learners to SCBOEI compared to the other two types of value is probably due to the nature of online education product that is highly functional by nature. Rewarding experience derived from functional benefits of the products further increase the identification with online education communities. Entertainment and other hedonic features of the online education products are of secondary importance.

Through social identity, value also asserts indirect effects on SCBOEI. In that, students who place importance on social responsibility, education quality, and positive development take a more active role in online education community by assuming the role of sharer. Sharing content online helps narrowing the education equality gap and better realize other sustainable development agenda. Expressing one’s social responsibilities in online education communities is determined by their sense of obligation for sustainable behaviors including SCBOEI. In turn, higher identification with online education communities also reinforces their sense of value as well.

To promote higher SCBOEI, policy makers and practitioners can use the proposed model in this study that combines Consumer Value Theory with Social Identity Theory. Higher identity to the online education communities improves understanding of sustainable consumption in online education and enhance the sense of responsibilities. It can be argued therefore, that social identity is the key factor of the model. Stakeholders in the online education industry should create the experience of learners that increase the sense of identification to the community by augmenting the awareness of social aspects of online education and by encouraging learners to be both consumers and providers of the content. As such, attention should be paid to factors and incentives that enhance the probability of students to shift their roles from content consumer to content sharer.

## Limitation of the Study and Suggestion for Future Research

The present study did not explore and examine the effects of various factors potentially influence SCBOEI including attitude toward sustainable consumption, social media, age, gender, education level, socio-economic status, and urbanization. For example, social media has a particular role in promoting online education (Kumar and Nanda, 2019). People with higher education can receive online education more (Kumar and Nanda, 2019). The online education industry in big cities is more developed than in small cities (Frick and Rodríguez-Pose, 2018). Online education products in big cities are more abundant, have more choices, and may have advantages in terms of price and access (Pappas et al., 2018). The convenience of online education increases the possibility of college students to choose online education over the traditional classroom mode (Sadeghi, 2019). However, students’ identification and emotional interaction, which are highly important to tackle complex topics and cope with learners’ problems, are still incomparable to (Atlam et al., 2021). Future studies should examine the effects of these factors on SCBOEI.

Geography is also a limitation of this study. Data collected in this study are mainly from China that has special socio-economic conditions comparing to other countries both pre and during COVID-19 period. Future research should also be conducted on other countries. Even in China, other scholars interested in the area should conduct their future work in students of other educational levels as well.

Other factors will affect sustainable consumption behavior. These factors may be sustainable consumption attitude, social media, age, gender, education level, family income. For example, social media has a particular role in promoting online education (Kumar and Nanda, 2019). People with higher education can receive online education more (Kumar and Nanda, 2019). The online education industry in big cities is more developed than in small cities (Frick and Rodríguez-Pose, 2018). China’s education policy has a noticeable impact on the online education industry (Chen et al., 2020). In this study, the socio-economic status of college students is not high. Therefore, in addition to these socio-economic factors, other factors may affect college students’ consumption behavior of purchasing online education in this study. These factors may be price and geographical location. Online education products in big cities are more abundant, have more choices, and may have advantages in price (Pappas et al., 2018). The convenience of online education increases the possibility of college students choosing online education (Sadeghi, 2019). However, online education’s identity and emotional transmission cannot compare with the traditional education model, which is a complex problem encountered by many college students in solving knowledge (Atlam et al., 2021). Furthermore, future research will summarize this finding according to different samples of different universities in different regions.

## Conclusion

In order to promote Sustainable Consumption Behavior in Online Education Industry (SCBOEI), the present study examine the factors that determine the intended behavior especially in social aspects of sustainable development using Consumer Value Theory in conjunction with Social Identity Theory. A survey was conducted with Chinese students mainly in the undergraduate level. Using Structural Equation Model as the analytical tool, the results show a critical influence of value and social on SCBOEI. Besides, values (functional, social, and emotional) also assert their influence on SCBOEI indirectly through social identity showing the causal chain relationship between the variables. Implications for both academics and practitioners were given highlighting on the importance of incrementation of social identity through value. Stronger identified members of online education communities tend to express more SCBOEI.

## Data Availability Statement

The datasets presented in this article are not readily available because they involve the interests of collaborators, as well as some privacy issues, and some data are confidential. However, further individual scholars or experts are welcome to request these datasets for academic references or other needs; requests to access these datasets should be directed to SJ, jiang680801@163.com.

## Author Contributions

SJ and RP: conceptualization and writing—original draft preparation. SJ, RP, and NJ: methodology, formal analysis, and writing—review and editing. All authors have read and agreed to the published version of the manuscript.

## Conflict of Interest

The authors declare that the research was conducted in the absence of any commercial or financial relationships that could be construed as a potential conflict of interest.

## Publisher’s Note

All claims expressed in this article are solely those of the authors and do not necessarily represent those of their affiliated organizations, or those of the publisher, the editors and the reviewers. Any product that may be evaluated in this article, or claim that may be made by its manufacturer, is not guaranteed or endorsed by the publisher.

## Appendix 1

### Sustainable Consumption Behavior in Online Education Industry

In the past year, I spent some time learning online and caring about sustainable development.

I prefer to buy shareable online education products.

I will engage in daily activities of caring for and safeguarding social equality.

### Functional Value

Online education products have consistent quality.

The online education products will be well made.

The online education products will be economical.

The online education products will offer value for money.

### Emotional Value

Buying the online education product would feel like making a good personal contribution to something better.

Buying the online education product would feel like the morally right thing.

Buying the online education product would make me feel like a better person.

### Social Value

Buying the online education product would help me to feel acceptable.

Buying the online education product would improve the way that I am perceived.

Buying the online education product would make a good impression on other people. Buying the online education product would give its owner social approval.

### Social Identity

I feel like a member of the online education industry.

I am a long-term consumer of online education.

I think other consumers of online education have similar interests and hobbies with me.

I agree with the values of the online education industry.

I agree that the online education industry can serve the image of the society.

I agree with the sharing concept advocated by the online education industry.
